# Reorganization of the imaging units in the context of the COVID-19 pandemic: experience of the Ibn Sina University Hospital in Rabat

**DOI:** 10.11604/pamj.supp.2020.35.2.23239

**Published:** 2020-05-11

**Authors:** Fatima Zahrae Laamrani, Aicha Chaibi, Nabil Moatassim Billah, Ittimade Nassar, Fatima zahrae Lasri, Amal Amrani Alaoui, Laila Jroundi, Naoufel Madani

**Affiliations:** 1 Emergency Radiology Depatrment, Ibn Sina Hospital, Rabat, Morocco; 2 Clinical Pharmacy Department, Medicine and Pharmacy Faculty, Mohamed V University, Rabat, Morocco; 3 Central Radiology Department, Ibn Sina Hospital, Rabat, Morocco; 4 Medical Emergencies Department, Nosocomial Infection Control Committee, Ibn Sina Hospital, Rabat, Morocco

**Keywords:** COVID-19, organisation, imaging

## Abstract

The global health system is currently facing the new SARS-COV 2 pandemy. This exceptional situation requires, from our African health systems, to reorganize and readapt the usual protocols when they were carried out before the crisis and/or their urgent implementation otherwise. As imaging is one of the pillars of the diagnosis of infection with this emerging virus, it was essential to rethink the imaging department organization so as to dedicate a unit to COVID-19 activity while maintaining the usual emergency activity within the Ibn Sina university hospital in Rabat. The protection of exposed personnel and the bio-cleaning of radiology equipment and rooms also became an evidence. The active involvement of the administration, the Clinical Pharmacy Department and the Nosocomial Infections Control Committee is a key to the success of this reorganization.

## Perspective

The global health system is currently facing the new SARS-COV 2 pandemic. This exceptional situation requires, from our African health systems, to reorganize and readapt the usual protocols and their urgent implementation. As imaging is one of the pillars of the diagnosis of infection with this emerging virus, it was essential to rethink the imaging department organization so as to dedicate a unit to COVID-19 activity while maintaining the usual emergency activity within the Ibn Sina university hospital in Rabat. The protection of exposed staff and the bio-cleaning of radiology equipment and rooms also became an evidence. The active involvement of the administration, the Clinical Pharmacy Department and the Nosocomial Infections Control Committee is a key to the success of this reorganization. In recent weeks, the imagery team of Ibn Sina University Hospital in Rabat (Morocco) has been confronted with a new activity, the COVID-19 activity [1-3]. The gradual increase in suspected or confirmed Covid cases in Morocco has made it possible to gradually set up an organization and a protocolization of all aspects related to “Covid” radiology, which we share through two aspects.

Organizational component: Ibn Sina University Hospital in Rabat normally has 800 hospital beds. The non-urgent activity (scheduled surgery, consultations and hospitalizations except emergencies) was suspended and the hospital was geographically divided into two activities with separate circuits: a dedicated “Covid” activity located in the basement and on the ground floor which includes the emergency reception service, the emergency radiology service, a second so-called central radiology service and several hospital units; a non-Covid activity located on the floor level and reserved for urgent non-Covid activities. The imaging activity, like all hospital activity, has been divided into: a Covid activity which is taken care of by the emergency radiology service which performs computed tomography (CT) and chest x-rays for suspected or confirmed patients infected with Covid-19 arriving from the emergency reception service or other hospital units. Any respiratory symptomatology that is not clinically proven is considered suspicious; a non-Covid activity carried out in the central radiology department, which is responsible for imaging other hospital patients. A clinical information collection form has been developed to facilitate the referral of patients receiving Covid imaging to chest x-ray or CT rooms. The indications for CT have been widened due to its high sensitivity, although its specificity is discussed [4].

Bio-cleaning, disinfection and Protection of personnel: bio-cleaning and protection of caregivers (doctors, radio handlers and support staff) working in radiology were not formalized in our hospital before the health crisis. In the urgency of the situation, the clinical pharmacy department, the nosocomial infection control committee and the radiology department set to work. In addition to a review of the recent literature on the contamination and contagiousness of SARS-COV 2, the effectiveness of the implementation of the protocols was conditioned by three essential factors: 1) The involvement of the personnel; 2) The effective application of the protocols:it was possible either by simulations or by its implementation when a Covid patient arrived in radiology; The operational hygiene team of the hospital, reporting to Nosocomial Infection Control Committee (NICC) and the Nursing Service, were of undeniable contribution; 3) The total acceptance of the administrative staff and the hospital pharmacy to all our requests in terms of organization, supplies of hygiene products and protective equipment. The available data in the literature prompted us to opt for draconian disinfection measures in an imaging environment considered to be a high-risk environment, among other things because of the confined nature of the radiology rooms. Studies conclude that SARS-Cov-2 persists on different surfaces and reveals that plastic and stainless steel offer greater stability to the virus [5,6]. As for airborne transmission, if no air sample taken from the isolation rooms of three patients with COVID-19 was positive in the study reported by Ong et al [7], the virus was detected on the air outlets, which could suggest a possible transmission of micro-aerosols contaminated by the air flows. Besides, more and more data tend to show the viability of the virus in aerosols for several hours, especially in confined spaces [8].

SARS-COV-2 being an enveloped virus, its inactivation can be effectively carried out by surface disinfection procedures with solutions containing 62-71% ethanol, 0.5% hydrogen peroxide or 0.1% sodium hypochlorite with minimum contact time of 15 minutes [9]. The EN 14476 standard for detergents/disinfectants is recommended for enveloped viruses and would by analogy inactivate SARS-CoV-2 [10-12]. The main drawback of these protocols is the limited number of examinations that can be performed per day since the imposed airborne disinfection requires 45 minutes between each examination, to which must be added 15 minutes of bio-cleaning. The airborne disinfection does not disinfect the air in the contaminated room (only ultraviolet rays have demonstrated their effectiveness) but it makes it possible to reach areas that are not easily accessible by surface disinfection techniques and thus limit the risk of persistence of a micro-reservoir pathogenic organisms. Properly performed bio-cleaning theoretically dispenses with an airborne disinfection [13]. We still opted for an airborne after each visit to the imaging room, on the one hand for its proven effectiveness in a pandemic context, but also to calm hygiene agents having to perform a manual bio-cleaning after airborne disinfection.

It should be noted that after more than a month of Covid activity, 260 scanners and 180 chest x-rays, no one of the emergency radiology department staff was contaminated. The protection of the radiology staff in contact with the patients is achieved by the installation of a specific kit including (gown, overblouse, FPP2 mask, charlotte, overshoes and gloves) and by a short training on its use, especially on dressing and undressing. Ultrasound in suspected or confirmed COVID-19 patients is conceived in two distinct situations: 1) Pulmonary ultrasound as part of screening or monitoring of lung lesions. It is currently in the research field and it is recommended that CT replace it whenever possible. Nevertheless, in the eventual absence of the scanner under our skies and in case of availability of expertise in pulmonary ultrasound, the latter is probably an interesting alternative. 2) Ultrasound other than pulmonary in the context for example of digestive, renal, vascular or other symptomatology associated with the classic symptomatology of Covid-19 infection or in the context of a complication. Rigorous protective measures must be implemented during an ultrasound due to an extended contact time [14-16]. The bio-cleaning protocol is carried out in the same way as in the CT or standard radiology room.

## Conclusion

We wanted to bring our experience in the reorganization of hospitals in general and of the Radiology department in particular while facing the Covid-19 pandemic. The aim is to facilitate and inspire our colleagues, brothers and sisters from the rest of our continent, for the organization of their own units if it has not already been set up. To each thing woe and good as they say. This exceptional situation forced us to improve hygiene practices in our hospitals and to implement protocols that normally should have existed before.
